# Particle alignment reliability in single particle electron cryomicroscopy: a general approach

**DOI:** 10.1038/srep21626

**Published:** 2016-02-22

**Authors:** J. Vargas, J. Otón, R. Marabini, J. M. Carazo, C. O. S. Sorzano

**Affiliations:** 1 National Center for Biotechnology (CSIC), c/Darwin, 3, Campus Universidad Autnoma, 28049 Cantoblanco, Madrid, Spain; 2 Bioengineering Lab. Univ. San Pablo CEU. Campus Urb. Monteprncipe s/n. 28668 Boadilla del Monte, Madrid, Spain; 3 Escuela Politécnica Superior, Universidad Autónoma de Madrid, Campus Universidad Autónoma, 28049 Cantoblanco, Madrid, Spain

## Abstract

Electron Microscopy is reaching new capabilities thanks to the combined effect of new technologies and new image processing methods. However, the reconstruction process is still complex, requiring many steps and elaborated optimization procedures. Therefore, the possibility to reach a wrong structure exists, justifying the need of robust statistical tests. In this work, we present a conceptually simple alignment test, which does not require tilt-pair images, to evaluate the alignment consistency between a set of projection images with respect to a given 3D density map. We test the approach on a number of problems in 3DEM, especially the ranking and evaluation of initial 3D volumes and high resolution 3D maps, where we show its usefulness in providing an objective evaluation for maps that have recently been subject to a strong controversy in the field. Additionally, this alignment statistical test can be linked to the early stages of structure solving of new complexes, streamlining the whole process.

The Electron Microscopy (EM) field is experiencing a very fast evolution in recent years. It has already been shown how new approaches, such as Direct Detector Devices and new image processing methods, have opened the way to obtaining quasi-atomic resolution for a large range of macromolecular complexes, including relatively small ones (less than 300 KDa). This expansion of the breadth of EM implies that it will naturally be used on new specimens for which very little complementary information may exist or, in general, specimens for which no much complementary information will be available. For these cases in particular, but for all cases in general, the development of new approaches to rank the consistency of a given map with respect to sets of EM images is of paramount importance. It is known that reconstruction procedures may be trapped into local minima, as it may happen when the provided initial map is not good enough or when the sample is affected by heterogeneity, among other problematic cases. Ways to check how likely these problematic situations may occur are strongly needed. Indeed, some recent structures of key biomedical specimens^1^ have been the subject of much controversy in the field (as exemplified in their corresponding replies^2^,^3^,^4^,^5^), clearly illustrating the vital need to incorporate rigorous and informative tests in EM reconstruction procedures.

Currently, alignment evaluation normally requires the analysis of pairs of particle images recorded at different tilt angles (tilt-pairs)^6^,^7^. These tilt-pairs based methods work comparing the difference between the calculated orientations among the non-tilted and tilted particles, with respect to the known tilting angle. This discrepancy is an indicator of the 3D map quality. However, tilt-pairs analysis requires to increase the amount of data to collect and process. Furthermore, in many occasions, the evaluation is performed retrospectively, and then, in these cases tilt pairs may not be available. Moreover, Beam Induced Movement may introduce an extra source of discrepancy or dispersion in the projection plot. Additionally, collecting high-resolution and high-quality tilt-pairs is, itself, a relatively challenging experiment, as very often drift and/or charging occur in the tilted images^8^.

In this work, we present a statistical methodology, which does not require tilt-pair images and therefore, it is of general applicability (including retrospective analysis). The approach can be used to evaluate the consistency between a raw 3D density map with respect to a set of 2D projection images, that were used to reconstruct that volume. The proposed method can also be applied to rank the alignment precision quality of a set of proposed 3D maps with respect to a set of projection images (or 2D class averages), also used in the reconstruction of the initial maps. Therefore, this approach is specially suited for the selection of a reliable initial map. Importantly, the proposed particle alignability approach gives information about the particle alignment precision but not about the particle alignment accuracy, as tilt-pair validation does. As a consequence, this method should be considered as a necessary but not sufficient test to evaluate the quality of a 3DEM map. Furthermore, this map quality test is performed using a specific alignment procedure, whose performance may depend on the sample images.

## Results

### Outline of the method

The objective of this work is to provide a statistical analysis, without using tilt-pairs, with the capability to provide objective information about the consistency between the reconstructed 3D map and a set of 2D projections (or 2D classes) used in the map reconstruction process. Our work is based on studying, for each experimental projection image, the weighted orientation distribution of their corresponding most similar map projections, according to a significant value. Note that these map projected images are obtained projecting the volume into a regular angular grid. We will refer to the map projected images in the following parts of this paper as reference images. Moreover, the similarity metric needed to quantify the likeness between the projection and reference images can be the well-known normalized correlation or any other different similarity metric, such as IMED^9^, for example. In this work, following^10^, we have used a probabilistic approach for the similarity or weight calculation between the projection and reference images, but other methods, such as RELION^11^ could also be used.

In order to analyze if a projection image is consistent with a 3D density map, we can study the weighted orientation dispersion, or clustering tendency, of their corresponding most similar reference images, according to a significant value. As illustrative examples, we may think first of a case where for each experimental projection image its most similar reference set is characterized by a very clustered angular spread, with a weight -or similarity value- that is very high at the cluster center and which decreases smoothly and quickly as we move away from this center. In this situation, the orientation determination of the projection images should be very reliable, and the final computed 3D map will likely be correct until a certain resolution value, that depends on the number and the angular distribution of the projected images that have entered into its calculation. At the other extreme, we may think of a situation in which for each projected image, the weighted orientation angular distribution of the set of most similar references (according to a certain significant value) is completely scattered. In that case we would be unable to assign a reliable orientation to each projection image, and the final 3D map could not possibly be correct.

In order to quantify these two critical scenarios, and cases in between, we have used a weighted clustering tendency parameter, inspired on the Hopkins statistic parameter. The Hopkins statistic parameter of a set of *M* points (*S*) is defined as^12^.


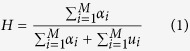


where *α*_*i*_ is the distance between point *i* and its respective closest point belonging to point set S, and *u*_*i*_ is the distance between point *i* and its respective closest point belonging to an equivalent uniform random distribution over the projection sphere and composed of the same number of points as point set S.

**Figure 1:**
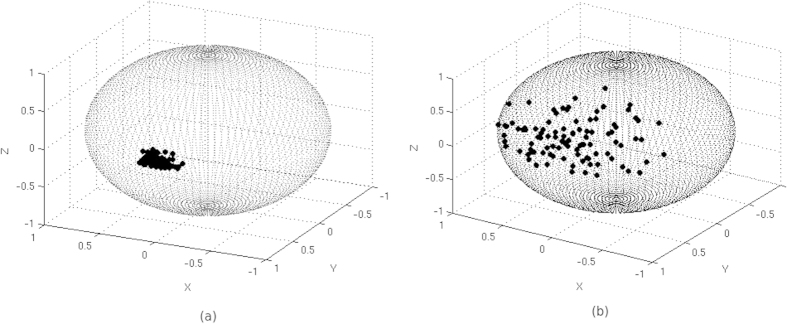
Examples of a clustered orientation distribution over the unit sphere (**a**) and a not clustered distribution (**b**).

This statistic examines whether objects in a dataset differ significantly from the assumption that they are uniformly distributed in a multidimensional space. Observe, that *H* provides us with important clustering information. A value close to zero means tightly clustered point set, while close to 0.5 corresponds to no clustering. In our case, we have to analyse the clustering tendency of points distributed over the unit sphere (orientation distribution). In Fig. 1 we show an example of a clustered orientation distribution over the unit sphere (a) and a not clustered one (b). In this work, we use the geodesic distance over the unit sphere as the metric to obtain the distance from one point *i* to its closest one, given as


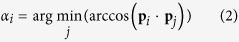


and for an equivalent uniform randomly distributed point set


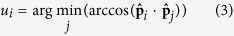


where 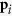 and 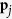 are an arbitrary point and its closest one, both belong to *S*, while 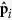 and 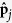 have the same meaning as 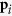 and 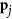, but belonging to a uniform random distribution on the projection sphere. Note that these vectors are unitary. Observe that in Eq. (2) and Eq. (3) there is no information about the weight or similarity value distribution. As explained above, it is desirable that this similarity or weight distribution is also structured; in other words, we want the closest points to have high and alike similarity values. Therefore, we define a weighted version of Eq. (2) and Eq. (3) as


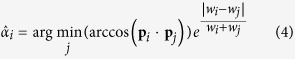



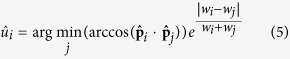


with 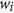 and 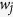 being the similarity or weight values of the corresponding *i* and *j* orientations. Note that in Eq. (5) we are using the same similarity values as in Eq. (4). Note that introducing weights in Expressions (4) and (5) is a key issue in order to provide strong robustness against noise to our alignment evaluation approach (please, see Supplementary Material for further information). Finally, we can define a weighted clustering tendency parameter as


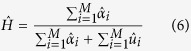


from Eq. (6) we can obtain a weighted clustering tendency parameter when the points are uniformly random distributed as


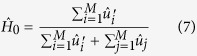


where 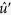 is a realization different from 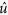 of the random process that scatters a set of projections on the unit sphere. From each projection image *k*, using Eq. (6) and Eq. (7) we can estimate their empirical distributions 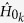 and 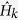 by using a Monte Carlo sampler (in our case, we sample 100 times). From these distributions, we determine the corresponding cumulative density functions as


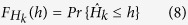



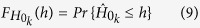


From Expressions (8) and (9), we define the inverse cumulative density functions, 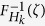 and 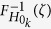, which for each percentile give us the corresponding 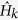 and 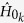 values. With these inverse density functions, we obtain, for each projection image, the clustering ratio as


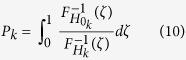


After we have computed 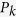 for all the projection images using Eq. (10), we can define a map consistency or quality parameter *Q* as


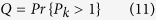


Using this volume consistency or alignment precision quality parameter *Q*, we establish that a volume is not reliable if 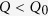, taking into account our alignment procedure, the significant value and angular sampling used, which are input parameters of the proposed approach. In our experiments, we chose 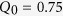 when using typical values of significant value and angular sampling of about 0.05 and 10 degrees, respectively. Note that in tilt-pairs validation approach it is established as angular validity criteria that at least 60% of the particles must show a single cluster^6^. This criteria implies a *Q*_0_ threshold of 0.8 (please see Supplementary Material for further details). However, from all the tests performed in our work, we have checked that there is a large number of cases where the 3DEM map is correct, at least at medium resolution, with *Q* values close to 0.75. Therefore, we have decided to set the *Q* threshold value to *Q*_0_ = 0.75.

**Figure 2:**
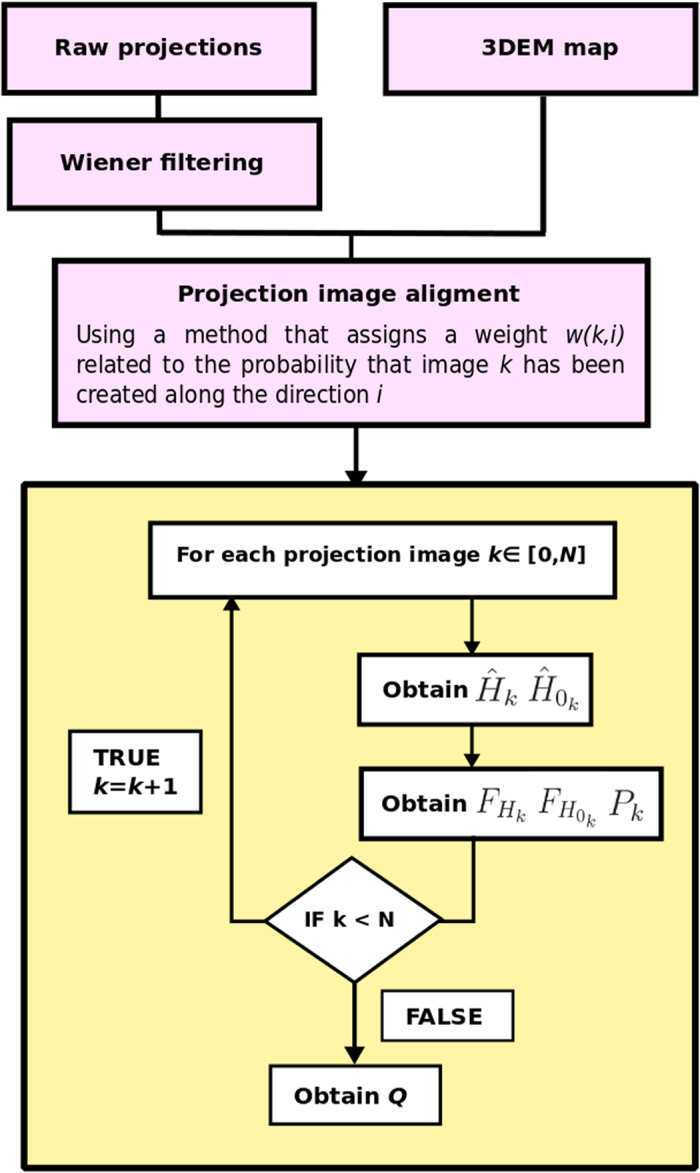
Diagram of the proposed alignment reliability approach.

Figure 2 presents a schematic flow diagram explaining how *P*/*Q* are calculated in practice. The inputs of the algorithm are the 3DEM map and the projection images used to reconstruct the map (or a smaller subset which sample uniformly the projection sphere). This input is firstly *CTF*-corrected by Wiener filtering^11^, where the *CTF* is obtained using Xmipp method^13^. Secondly, a projection image alignment process is done, using any method that assigns to each projection image the set of its most likely volume orientations (providing also the respective weights or likelihoods), according to a significant value. After this, 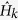 and 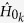 are obtained for each projection image using Eq. (6) and Eq. (7) and, then, *P*_*k*_ through Eq. (10). This process is repeated for all images and, finally, *Q* is calculated using Eq. (11). The proposed map alignment precision evaluation process should be performed with the same projection images used to reconstruct the map or, alternatively, using a smaller subset randomly selected which sample conveniently the projection sphere. Note that if the volume is reconstructed with raw projection images but the alignment evaluation approach is performed with class averages, the resultant *P*/*Q* values will be overestimated. These overestimated results are not mainly due to the higher *SNR* of the class averages with respect to the raw projection ones, but the explanation is because class averages have significantly higher spatial coherence than raw projection images, as the 2D/3D alignment process is never perfect.

### Application examples

We envision two types of situations in which this evaluation will be relevant: (1) Submission of a map to PDB/EMDB, and (2) Guidance of the reconstruction process itself. We will provide examples of these two cases: (1) Clearly establishing which of two recent reconstructions of the HIV-1 Env trimer is more in agreement with the experimental data and, (2) Guiding the 3D reconstruction process by selecting among multiple options those suitable initial models which agree better with the images used to reconstruct these maps.

### Evaluating controversial maps

We will now apply our proposed alignment precision test to the ranking of two conflicting maps of the HIV-1 trimer which were the subject of a recent controversy in Proceedings of the US National Academy of Science^1^,^2^,^3^,^4^,^5^. We aim at quantitatively evaluating which of the two maps shows a better alignment precision, using our alignment procedure, with each of the two experimental data sets. In this way, we retrieved the two corresponding maps from EMDB 5447^1^ and EMDB 2484^14^, as well as, their initial experimental images from EMPIAR 10008 and EMPIAR 10004, respectively; for the evaluation we used approximately 1000 particles randomly selected from the two experimental data sets, as well as the corresponding EMDB maps. We used only 1000 raw images selected randomly instead of the whole set of images because 1000 projection images are enough to provide a good sampling of the projection sphere.

**Figure 3:**
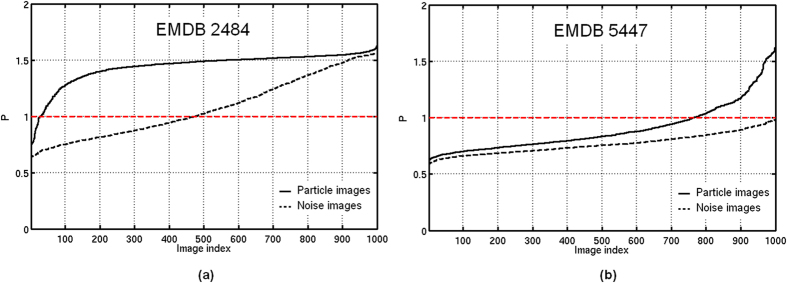
Clustering ratio *P* obtained when confronting EMDB 2484 map (**a**) and EMDB 5447 (**b**) map with their respective projection images used in Bartesaghi *et al.* work^14^ and Mao *et al.* work^1^ (solid curves), and using pure noise images picked directly from their own micrographs (dashed curves), respectively.

**Figure 4:**
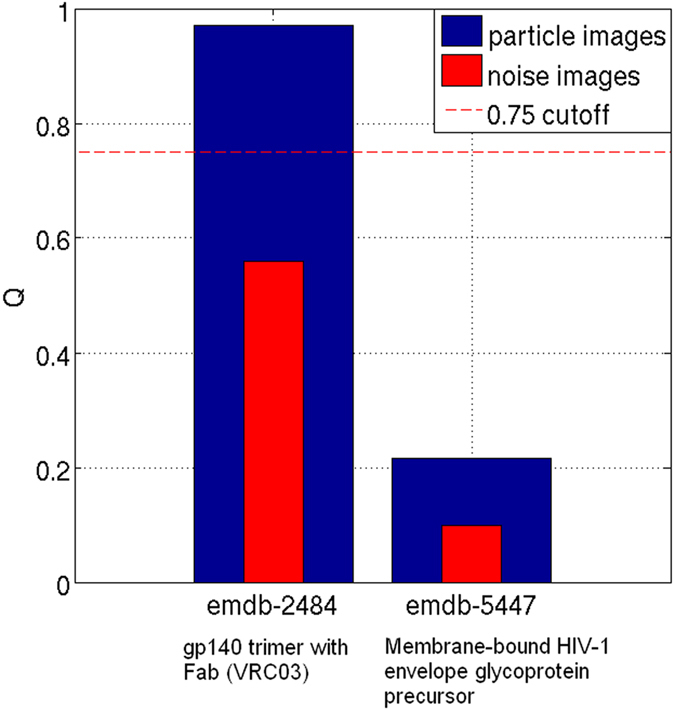
Obtained *Q* values when EMDB 2484 and EMDB 5447 HIV maps were confronted with the particles deposited by Bartesagui and colleagues (EMPIAR 10008) and by Mao and colleagues (EMPIAR 10004). Additionally, we show in red the respective *Q* values obtained when we confronted EMDB 2484 and EMDB 5447 maps with pure noise subimages picked manually from both Bartesagui’s and Mao’s micrographs respectively.

For the first test, the experimental EM images corresponding to Bartesaghi *et al.* work^14^ were confronted to their proposed map (EMDB 2484), as well as, the projection images from Mao *et al.* work^1^ were confronted to their own map (EMDB 5447). The resulting clustering ratios *P* are shown in Fig. 3(a,b), respectively. Additionally, we have also obtained the respective *P* values when EMDB 2484 and EMDB 5447 maps are confronted with pure noise subimages picked manually from their own micrographs. As can be seen from Fig. 3, the clustering ratio *P* plot obtained using Bartesaghi’s data and map (a) is significantly higher (and higher compared to *P* values obtained by pure noise images) than when using the Mao’s data and map (b). Additionally, from this data, we computed the respective global quality *Q* values corresponding to values of 0.98 for Bartesaghi’s map and of 0.23 for Mao’s map. As further explained in previous sections, *Q* values close to 1 correspond to reconstructions computed from particles orientated with high precision, while a 3D map characterized by a *Q* value close to 0 is expected to come from randomly orientated particles (note that a more quantitative relationship is provided in Supplementary Material). Clearly, Bartesaghi’s map is in good agreement with their input experimental images, while the precision of the alignment of Maos data is low, at least when our alignment procedure is used. These results are shown in Fig. 4. Additionally, we have also obtained the respective *Q* values when EMDB 2484 and EMDB 5447 maps are confronted with pure noise subimages picked manually from their own initial micrographs, and we are presenting these results on Fig. 4 in red color. In the case of EMDB 2484, the *Q* value and *P* curve obtained when this map is confronted with its own data are high and significantly different to the ones obtained using pure noise images. Therefore, this map can represent a correct map according to our used alignment procedure. However, when the map EMDB 5447 is confronted with its own data, the resultant *Q* and *P* values are very low. Therefore, this map cannot represent a trustful reconstruction at the resolution reported by the authors, at least when our alignment procedure is used. Consequently, the analyses performed so far provides useful information to the question of whether the two maps are in agreement with their own experimental images. Indeed, the result is very clear, with a *Q* value of 0.98 for Bartesaghi’s pair of map and particles, compared with a value of only 0.23 for the corresponding pair from Mao’s data.

**Figure 5:**
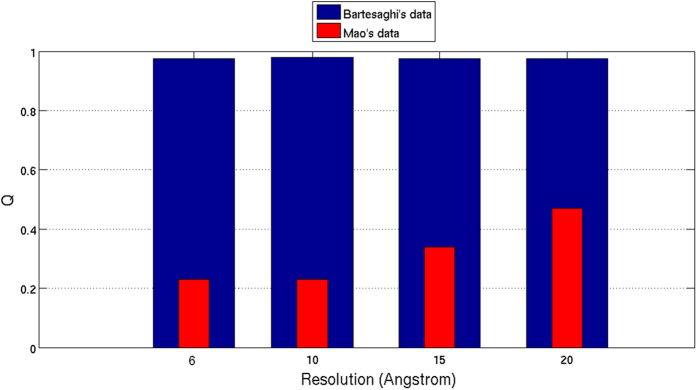
Obtained *Q* values when the Bartesaghi’s and Mao’s maps (EMDB 2484 and EMDB 5447) filtered at resolutions of 6, 10, 15 and 20 Å were confronted with the particles deposited by Bartesaghi and colleagues (EMPIAR 10008) (blue bars) and by Mao and colleagues (EMPIAR 10004) (red bars), respectively.

Finally, in order to analyse the effect of filtering the maps at different resolutions on the results provided by our proposed alignment reliability approach, we have obtained *Q* values when confronting both Bartesaghi’s and Mao’s particle images against their respective maps (EMDB 2484 and EMDB 5447) filtered at resolutions of 6, 10, 15 and 20 Å. The results are shown in Fig. 5, where blue and red bars correspond to Bartesaghi’s and Mao’s data. As can be seen from Fig. 5, the proposed approach recovers higher *Q* values at lower resolutions. Bartesaghi’s results present slightly differences at different resolutions, while Mao’s results show notable differences. This fact indicates that Mao’s map, low-pass filtered at 6, 10, 15 and 20 Å, has not the capacity to align its own data, at least using our alignment procedure. When this map is low-pass filtered to 10 Å the *Q* values start to increase.

### Guiding the reconstruction process

Nowadays cryoEM provides an unique way to study macromolecular complexes, requiring small sample volumes and relatively low protein concentrations, while being able to handle conformational mixtures. A simple estimation of the human interactome may suggest close to 150,000 interactions (using the STRING Data Base with the threshold set at 0.4), while the current number of corresponding complexes in PDB is less than 5,000. Moreover, many of these complexes represent only partially the structure of the interacting partners. In this context, it is clear that, in many cases, the cryoEM map reconstruction process might not benefit from any prior experimental structural knowledge. However, a number of reconstruction steps in 3DEM involves local optimizers, for which the initial value (the initial map), used for refinement through an iterative process is important. One typical procedure in the field, when no prior information is available, involves generating several initial maps that have been iteratively refined from initial 3D volumes obtained by randomly assigning class average orientations, followed by careful analysis looking for consistency. Thus we are often confronted by the need to choose an initial map without any clear guidance about how to proceed, or even the likelihood that any of them is correct. Motivated by this fact, we have explored the use of the here presented alignment evaluation approach to help in the initial map selection problem. To this end we have used the proposed approach in two experimental cases: GroEL and eukaryotic ribosome complexes.

### GroEL

**Figure 6:**
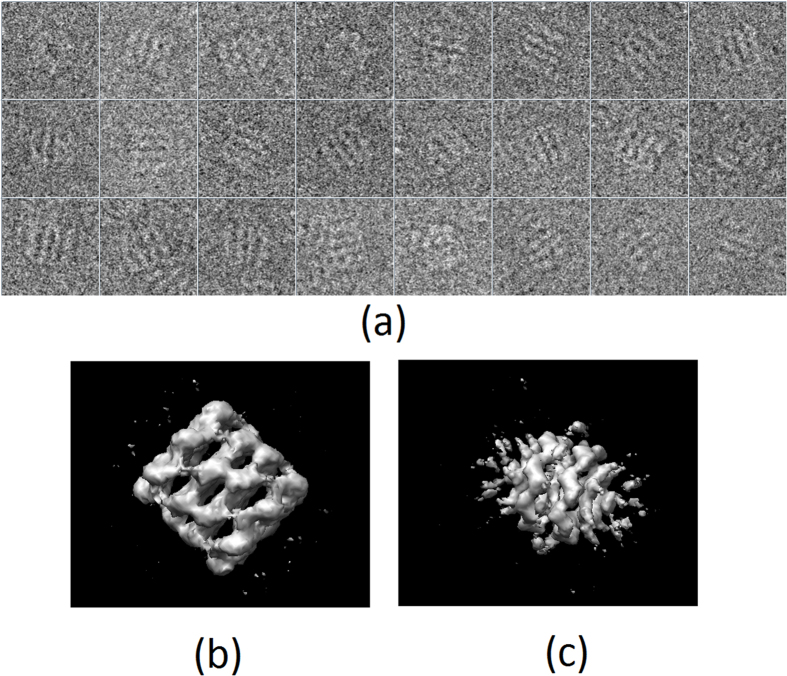
Some of the 2D projection images used for the evaluation (**a**) of the visually “correct” (**b**) and “not correct” (**c**) maps obtained by RANSAC initial volume determination approach.

GroEL^15^ is considered to be a very difficult case for blind initial map determination algorithms, as the top and side views have similar size and it is difficult to automatically decide which is the side and which the top view. Indeed these methods may get stucked into a local minimum, providing wrong 3D low resolution maps. We used the GroEL dataset publicly available as the tutorial of EMAN2 (http://blake.bcm.edu/emanwiki/Ws2011/Eman2)^16^, composed by 26 micrographs of size 4082 × 6278 pixels. The sampling rate was 2.10 Å/pixel and the microscope voltage 200 kV. From this dataset, we detected 4,123 particles of size 128 × 128 px, using the methods presented in^17^ and^18^, and 16 classes were determined using CL2D^19^. After this processing, we used RANSAC initial map determination approach^20^, which provided us with ten different maps. From this initial map set, we picked up two, one that clearly was not a correct initial map for GroEL, and another one that appeared to be correct (up to a certain spatial resolution). After that, we randomly selected a projection image subset composed by 1000 images and ran the proposed evaluation approach using the two selected initial maps. As input parameters of the proposed method we used a significant value of 0.05 and an angular sampling of 5 degrees. In Fig. 6, we show some of the 2D projection images that have been used (a) and the two maps obtained by RANSAC, the “correct” one (b) and the “incorrect” one (c). From Fig. 6(b,c), it is very clear that map (b) is a “correct” one, while (c) is not.

**Figure 7:**
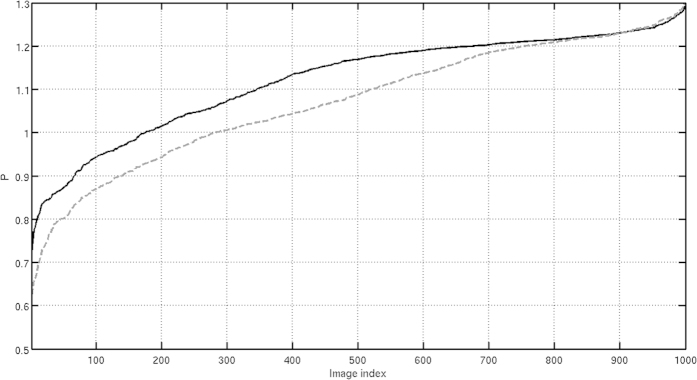
Clustering ratio *P* corresponding to the different projection images when we use the visually “correct” volume (black curve) and the “incorrect” one (dashed gray curve).

We have run the proposed evaluation approach with these 2D projection images and maps; in Fig. 7 we show the *P*_*k*_ obtained for the “correct” volume (solid black curve) and for the “incorrect” one (dashed gray curve). Additionally, we have obtained quality parameters *Q* of 0.70 and 0.82 for the “incorrect” and “correct” models, respectively. Observe that the “correct” volume has a *Q* value higher than our threshold acceptance value while the “incorrect” one has a lower value.

### Eukaryotic ribosome

**Figure 8:**
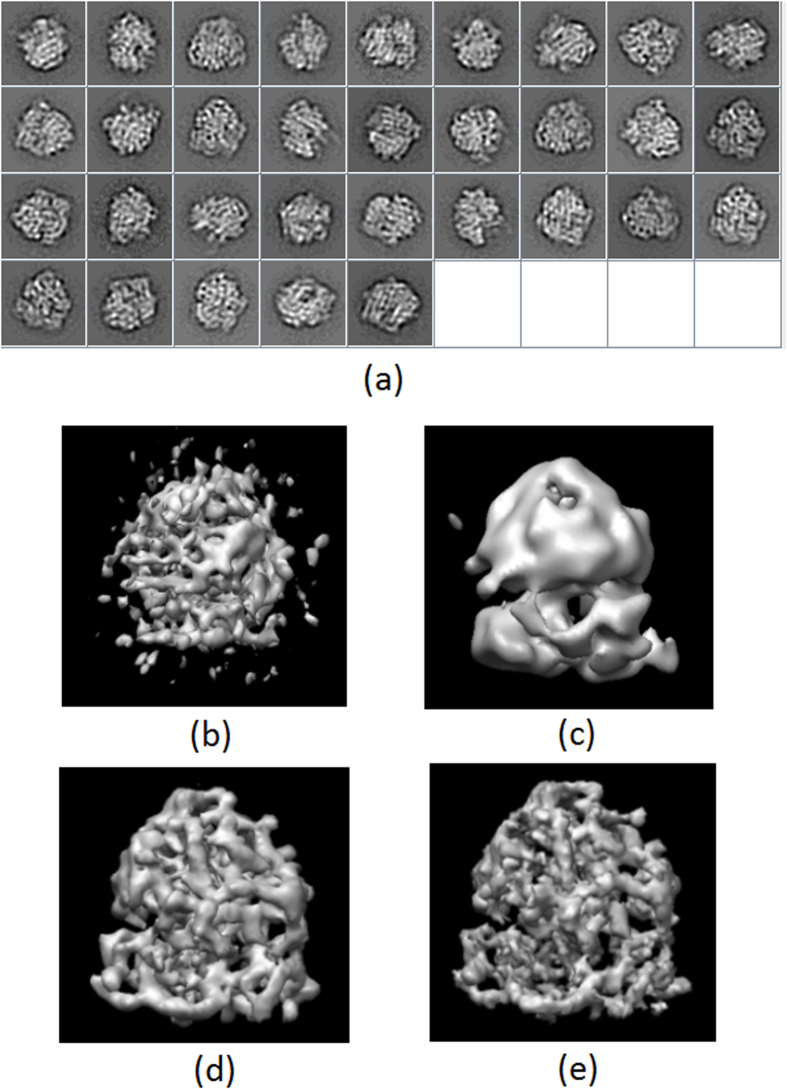
2D class averages of the Eukaryotic ribosome (**a**) and different selected initial maps computed by RANSAC (**b**,**c**) and Significant (**d**,**e**).

In our second experiment, we have used the eukaryotic ribosome images obtained from the EMDB test image data set (http://www.ebi.ac.uk/pdbe/emdb/test_data.html), and originally used in the work of^21^. We first obtained 32 2D class averages using CL2D^19^ from 5,000 cryo-EM projections. Then, we performed blind initial map determination using RANSAC^20^ and Significant^10^. After that, we selected two maps computed by RANSAC and other two by Significant, and we run our proposed alignment evaluation approach using the 2D classes and these maps. In Fig. 8 we show the classes (a) and the different maps selected by RANSAC (b–c) and Significant (d–e). As can be seen from Fig. 8, the map shown in Fig. 8(b) is not reliable, while the rest (c–e) are.

**Figure 9:**
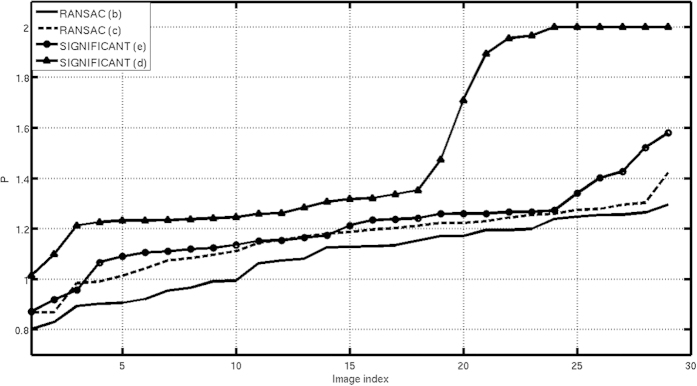
P index for the different projection images when we use the different volumes shown in 8 (b–e).

In Fig. 9 we show the results obtained after applying our proposed evaluation approach to the 2D projection class averages and to the reconstructed maps shown in Fig. 8. We have used a significance value of 0.05 and an angular sampling of 10 degrees. We can see clearly from Fig. 9 that the “incorrect” map has lower *P* values, for all the images, than the rest of the maps. Additionally, we have obtained the map quality parameters for all of these maps. The *Q* results for the maps shown in Fig. 8(b–e) are of 0.69, 1, 0.83 and 0.76, respectively. Observe that the “correct” maps have high *Q* values, while the “incorrect” one has a very low one.

## Discussion

The idea behind our approach is conceptually simple. It is based on the notion that for a given map to be compatible with a set of images, and in the absence of symmetries, most experimental images are expected to contribute to the map from one viewing direction, or, considering noise, from a narrow range of directions. In other words, that the distribution of likely orientations of an experimental image with respect to the map is clustered, as opposed to random. Naturally, symmetries have to be appropriately incorporated, as it is the case that some indistinguishable viewing directions for a certain map can also exist. Note that this simple concept of directionality clustering has been previously used when attaching a measure of quality to a map^7^,^22^, and that the main contribution of this work is to gather these previous observations into a formal approach able to quantitatively rank the likelihood of maps when tested against the experimental images without requiring any additional data (like, for instance, tilt pairs) and using the new concept of weighted directionality clustering. The input of the proposed alignment reliability method is a raw 3DEM map and the 2D projection images used to reconstruct the map (or a smaller subset which sample uniformly the projection sphere). Note that other “mixing” experiments should be used with caution as they can increase the chance of getting outside of the theoretical framework of our approach, and then, of producing overinterpretations and pitfalls.

Ideally, we want an approach that can validate the alignment precision of projection images (or of a large enough set) in situations of very low *SNR*. In these noisy situations, if the alignment is correct for a large percentage of the particles, obtaining a good map is typically only a matter of the number of the projection images used. In order to show the behaviour of our proposed approach in very noisy cases, we have performed different simulations presented in Supplementary Material. As can be seen from Figure S.2 in Supplementary Material, the *Q* parameter is approximately independent of the *SNR*. This can be easily understood taking into account that in our approach we use weighted clustering tendency parameters. Therefore, the alignment weights -or similarity values- play an important role in our alignment reliability approach. In our case, we bias the Hopkins cluster tendency parameter (*H*) by the similarity of the alignment weight distribution. Thus, in cases where the angular distribution is approximately randomly distributed, but the similarity or alignment weight distribution is structured and clustered, it will still produce good clustering ratio values. In other words, our alignment method is doing a good job dealing with noise, so that the clustering structure of the weights they produce is very robust against the noise. However, employing pure noise images does not necessarily imply zero or very low *Q* values as the noise might not be white and then it can present some degree of spatial coherence and therefore of alignability. As a consequence, a high *Q* value does not necessarily imply a good reconstruction or a correct map at a given resolution. However low *P*/*Q* values inevitably means a low quality or incorrect map, at least for the alignment procedure used in this test. Therefore, this method has to be thought as a necessary but not sufficient 3DEM quality check or test where low *Q*/*P* values can alert cryoEM practitioners to potential problems and indicate the need for improvement of analysis procedures or for additional data corroborating the map.

However, it is important to note that our proposed *Q* and *P* parameters say nothing about map resolution because the relevant information to align the projection images correctly comes from the low and medium resolution range. It is also important to mention that this alignment reliability test is not absolute in the sense that important information, such as the handedness of the map, can not be determined, so that additional methods must be used for this purpose, such as tilt-pairs. We have shown that around 1000 images are enough to perform a map alignment evaluation using the proposed approach. This opens the possibility of suggesting, as part of the EMDB submission process, the deposition of around 1000 raw randomly selected projection images. In short, *P* and *Q* parameters provide an objective measure of both the particle alignment precision quality and the proportion of particles with well-determined orientation parameters with respect to a given map, respectively. Acceptable *Q*/*P* values are a desirable but not sufficient condition for a map to be classified as correct. On the other hand, unacceptable *Q*/*P* is an indicator of potential problems and point out the need for improvement of analysis procedures or for additional data corroborating the map, as tilt-pairs. These parameters do not provide relevant information about the resolution and handedness of the map. Additionally, other key image parameters, such as defocus or magnification, are not considered. Therefore, these indicators alone have not enough information for a complete map validation in terms of deciding the absolute correctness of a cryo-EM map. An issue left for future exploration is the possibility of using the shape of *P* plots to analyze the occurrence of inhomogeneous datasets or the presence of sets of particles with very different quality.

The algorithm is available as part of the software suite Xmipp (http://xmipp.cnb.csic.es) and Scipion (http://scipion.cnb.csic.es) under the name “validation_nontilt”.

## Additional Information

**How to cite this article**: Vargas, J. *et al.* Particle alignment reliability in single particle electron cryomicroscopy: a general approach. *Sci. Rep.* **6**, 21626; doi: 10.1038/srep21626 (2016).

## Supplementary Material

Supplementary Information
