# Supplementary material for: Particle alignment reliability in single particle electron cryomicroscopy: a general approach
Source: Sci Rep. 2016 Feb 22;6:21626. doi: 10.1038/srep21626 (PMC4761946; doi:10.1038/srep21626)
Supplement: Supplementary Information [file srep21626-s1.pdf]

Supplementary Material for Particle alignment reliability in single particle electron cryomicroscopy: a general approach by Vargas et al.

J. Vargas, J. Otón, R. Marabini, J. M. Carazo & C. O. S. Sorzano

1. Relation between Q and the Signal to Noise Ratio of the raw projection images

An important question about the proposed method is the relation between Q and the signal-to-noise ratio (*SNR*) of the raw projection images. In order to analyze this relation, we have performed several simulations. For these simulations we have used an asymmetric structure obtained from the Protein Data Bank with code (PDB id:4MAO). In Figure S.1 we show an image of the resultant 4MAO density map at 2.60 Å resolution, which we will refer as "Phantom map".

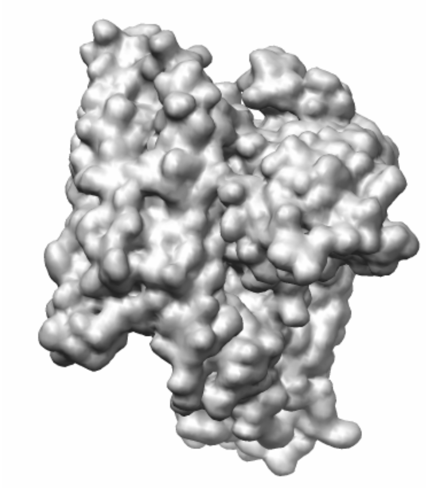

Figure S.1 View of 4MAO density map used in the simulations.

From this Phantom map, we obtained three different sets of around 1000 images by projecting the map at uniformly distributed orientations (covering completely the projection sphere) in three different *SNR* situations: without noise, first, and then with *SNR* of 0.1 and 0.01.We aligned these sets using Significant[9], obtaining three different

files with the angular and weight information of each projection image, and with these files we run our proposed orientation evaluation approach. Additionally, for the three different cases presented above, we modified the orientation of 20%, 40% and 60% of the projection images by uniformly assigning random orientations, and we run again our proposed method. From these different projection images and orientation files, we reconstructed the different 3D maps, and these maps were compared with the Phantom map using the Fourier Shell Correlation (*FSC*). *FSC* and *Q* results are shown in Figure S.2.

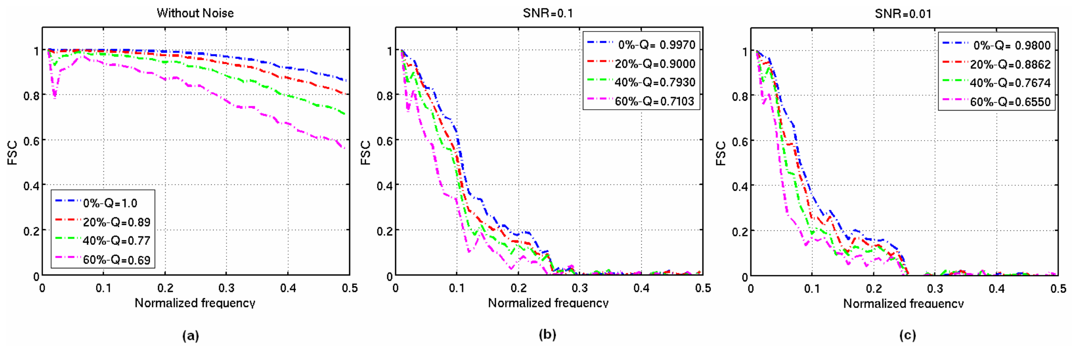

Figure S.2 *FSC* curves and *Q* values obtained from the data for different simulated noise cases, without noise (a), *SNR*=0.1 (b) and *SNR*=0.01 (c), when different percentages of particles are misaligned.

As can be seen from in the boxes included in each of the Figures S.2, the same % of misaligned particles produces very similar value of *Q*, therefore, we can conclude that *Q* is approximately independent of the *SNR*. Note that this is not the case of the *FSC* curves. This can be easily understood taking into account that in our approach we use weighted clustering tendency parameters (please see Expressions (4-5) and (7) in the manuscript). Therefore, alignment weights or similarity values play an important role in our alignment reliability method. In this way, we bias the Hopkins cluster tendency parameter (*H*) by the similarity or alignment weight distribution, and then, cases where the angular distribution is approximately randomly distributed, but the similarity or

alignment weight distribution is structured and clustered, will produce good clustering ratio values (Expression (10) in the manuscript). In other words, alignment methods are doing a good job dealing with noise, so that the clustering structure of the weights they produce is very robust against the noise. Additionally, observe that when *m* % projection particles are misaligned (with *m*=20, 40, and 60%) the obtained *Q* values are approximately 0.9, 0.8 and 0.7, respectively. In the main text we indicate that there is a direct relationship between *Q* and *m*, and in the following, we are going to derive this precise relationship. The simplest relationship would be that for a given *Q*, the percentage of misaligned particles would be 1-*Q*. However, this is not the case because when comparing two orientation sets with random uniform distributions (one coming from the random particle orientations and the other from the noise references), the respective $\hat{H}_k$ and $\hat{H}_{0k}$ will be both close to 0.5. However, due to small sample size effect and because $\hat{H}_k$ and $\hat{H}_{0k}$ comes from independent random events, we will have that since both $\hat{H}_k$ and $\hat{H}_{0k}$ are close to 0.5, in 50% of the cases we will have that $\hat{H}_k$ will be slightly higher that $\hat{H}_{0k}$, and therefore, P_k will be larger than 1 in these 50% of noisy cases. Taken into account this consideration, the *Q* parameter provided by our proposed alignment evaluation approach can be related with the detected percentage of misalignment particles (*N*) by the following expression

$$N = 2 \cdot (1 - Q) \times 100 \qquad \text{(S.1)}$$

In Table S.1, we show the respective *Q* and *N* values obtained in the different cases presented in Figure S.2, where *m* represents the percentage of manually misaligned particles.

m (%)	no noise		SNR=0.1		SNR=0.01	
	Q	N	Q	N	Q	N
0	1.0	0	1.0	0	0.98	4
20	0.89	22	0.90	20	0.89	22
40	0.77	46	0.79	42	0.77	46
60	0.69	62	0.71	58	0.66	68

Table S.1 Obtained Q and N values for simulations in the no noise and noisy cases when the orientation of different percentage of particles (m=0%, 20%, 40% and 60%) are set randomly.

As can be seen from Table S.1, there is a very good agreement between N and m parameters. Additionally, in Figure S.3 we show the 3D maps reconstructed from particles without noise, with SNR = 0.1 and SNR = 0.01, when particles are perfectly aligned (Figure S.3 (a), (b) and (c) respectively), on one hand, and when we modified the orientation of 60% of the projection images by random orientations (Figure S.3 (d), (e) and (f) respectively), on the other hand. These maps are low pass filtered at a cut-off resolution of 10Å. The map shown in Figure S.3(a) corresponds to the reconstruction from particles without noise and without misaligned orientations, and therefore is used as reference (note that it is essentially identical to the Phantom map to a resolution of 10Å).

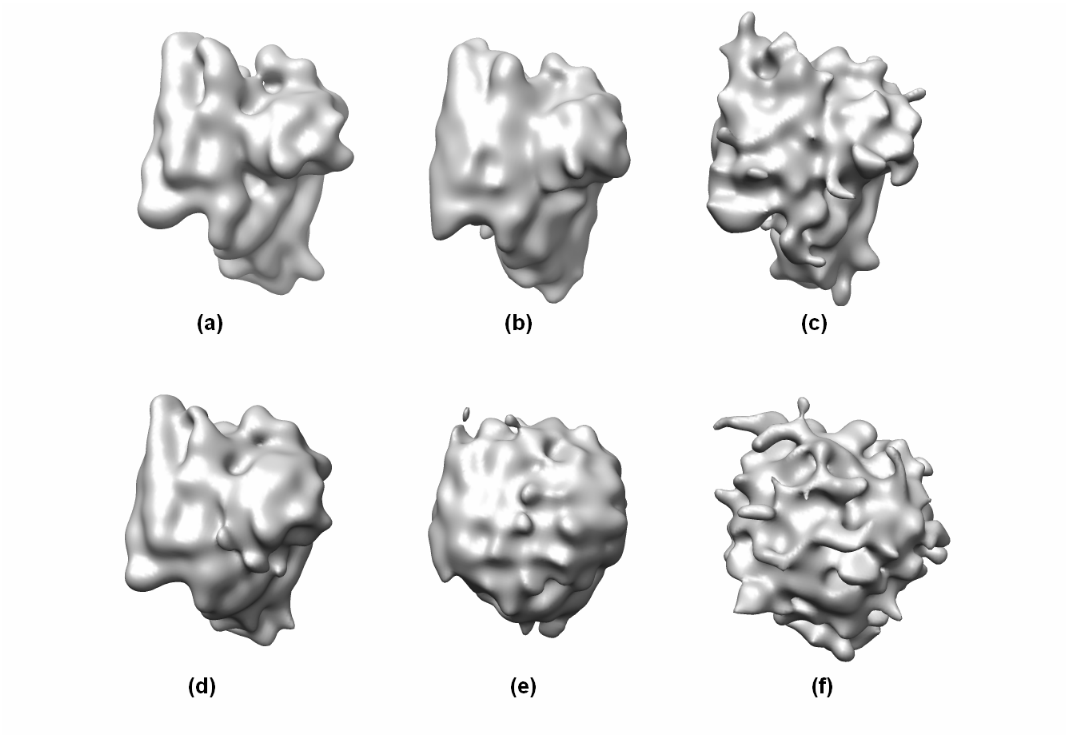

Figure S.3 Maps reconstructed from simulated perfectly aligned particles without noise (a), with *SNR*=0.1 (b) and *SNR*=0.01 (c), and then when 60% of the projections are misaligned for the three cases of without noise (d), with *SNR*=0.1 (e) and *SNR*=0.01 (f).

As can be seen from Figure S.3, maps (a), (b) and (c), which have *Q* values of 1.0, 0.997 and 0.98, respectively, are visually similar. On the other hand, *Q* values for maps (d), (e) and (f) are much lower, of 0.69, 0.71 and 0.65, respectively, clearly indicating that a significant percentage of particle images are not well aligned. Indeed, the accuracy of the determination of this percentage of misaligned particles is quite high, as shown in Table S.1.

Let us now concentrate on another important issue related to the practical use of *Q*. This is that, depending on the *SNR* of the images, the same % of misaligned particle images can produce a better or worse map, further indicating that *Q* is evaluating the consistency of angular alignment, but not other quality measures. Indeed, note that the map corresponding to Figure S.3(d) has a *Q* value (*Q*=0.69) relatively low, below our

threshold, although the map is visually correct. Further note that this map has been reconstructed from a particle set in which 60% of the projection images were misaligned. Therefore, it is normal that our alignment evaluation approach provides a low Q value. Additionally, the good shape of this map is just because as the projection images are not affected by noise, the resultant well-aligned 40% projection images are sufficient to reconstruct a correct volume. However, when noise has been added to the projection images (Figures S.3 (e) and (f)), this is not the case. Moreover, for the sake of clarity, we show in Figure S.4 the reconstructed volumes, low-pass filtered to 10Å resolution, in two cases in which the obtained Q values are close to our threshold Q_0=0.75, corresponding to values Q=0.79 and Q=0.77 (Please see Table S.1 to identify these volumes).

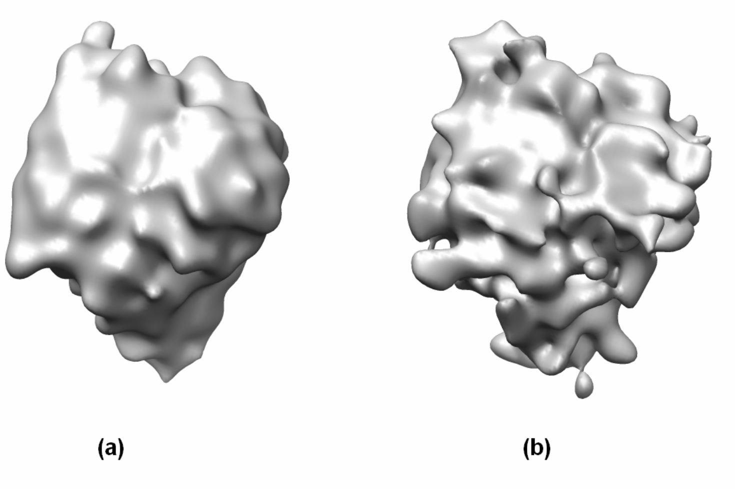

Figure S.4 Reconstructed volumes from simulated particles with SNR=0.1 (a) and SNR=0.01 (b) and with 60% of the projections images misaligned.

.

As can be seen from Figure S.4, the two volumes resemble one another visually. Finally, we have repeated the simulation when the *SNR* of the projection images is of 0.005 and without misaligned projection images. In this case, the resultant Q is of 0.98. In Figure S.5 we show the resultant reconstructed volume.

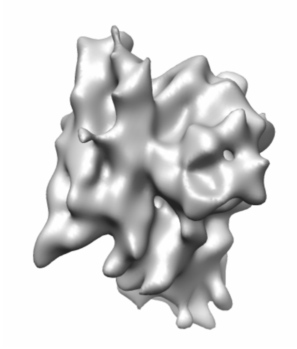

Figure S.5 Reconstructed volume from simulated particles with SNR=0.005 without misaligned projection images. Observe that this is a situation that normally would not occur.

2. Mixing experiments for HIV-1 maps

We have studied the behaviour of Q in hybrid cases, where we have confronted Bartesaghi's and Mao's particles with additional HIV maps, corresponding to EMDB 2484, EMDB 5447, EMDB 5018, EMDB 5019, EMDB 5462 and EMDB 5418, all filtered to a common resolution of 20 Å. Certainly, these comparisons are conceptually very appealing, but they should be taken with a lot of precaution; indeed, as a general rule, they should not to be done. Our alignment precision approach has to be applied using a raw 3DEM map together with a set of particles that were used in the volume reconstruction. Clearly, any mixing experiment can only increase the chance of getting outside of our theoretical framework. Therefore, the drawn conclusions must be taken with a lot of care, extracting only very general conclusions and avoiding elaborated over interpretations. Furthermore, the specimens are not exactly the same, since in the case of Bartesaghi the HIV trimer has Fabs attached, while Mao's specimen has not; naturally, the molecular mass of the two types of complexes is different, being in the range of 0.67 and 0.45 MDaltons, respectively. Moreover, some of the other HIV maps listed above have or not have antibodies attached. All these results are presented in Figure S.6. In this figure the blue and red bars represent the obtained values when particle and noise images are confronted with the different maps. The results achieved when the

Bartesaghi’s and Mao’s images are confronted with their respective maps filtered (EMDB 2484 and EMDB 5447) appear underlined.

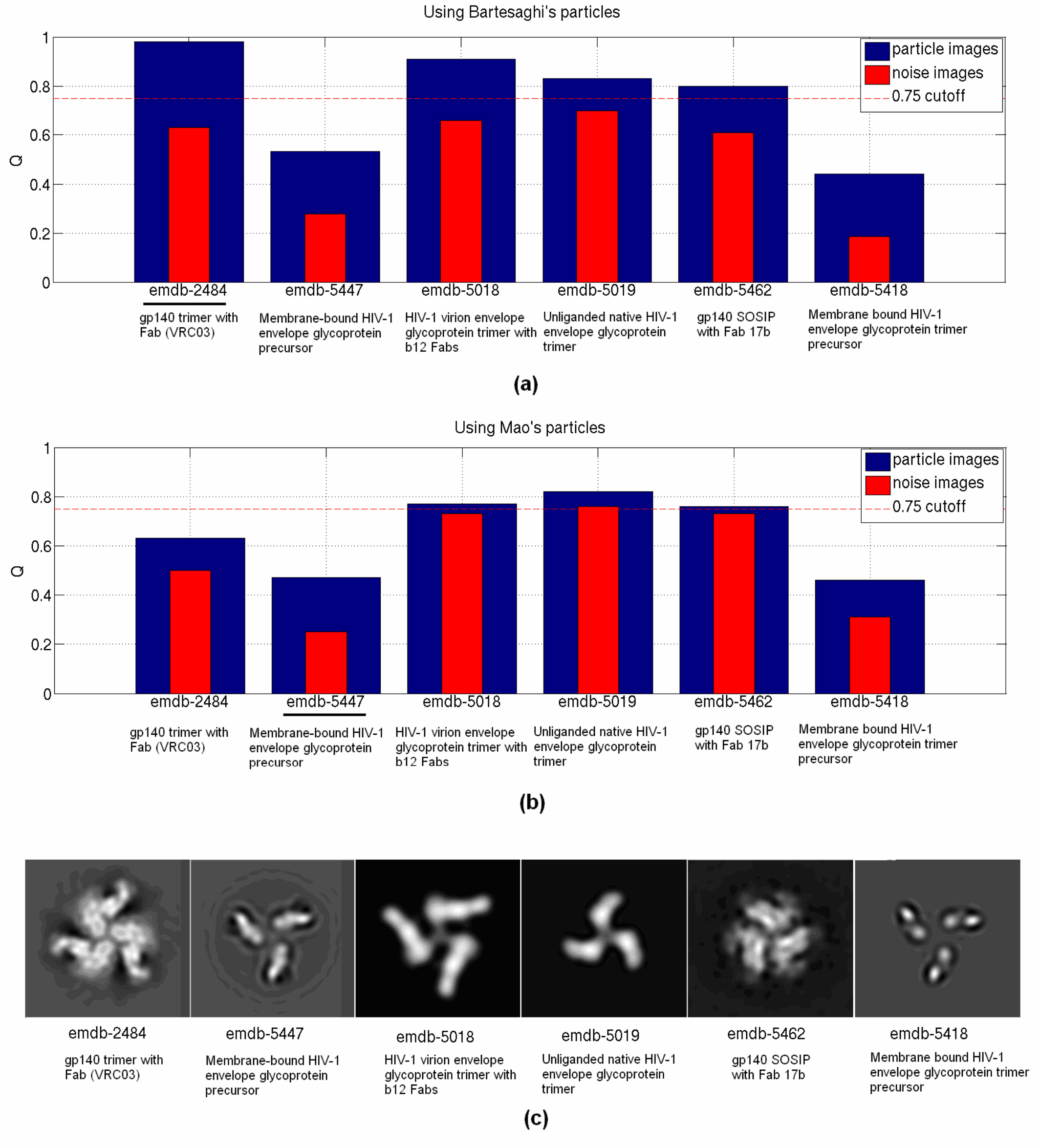

Figure S.6 Obtained Q values when different HIV maps with and without antibodies attached were confronted with the particles deposited by Bartesaghi and colleagues (EMPIAR 10008) (a) and by Mao and colleagues (EMPIAR 10004) (b). The Q values obtained when the Bartesaghi’s and Mao’s particles are confronted with their respective maps (EMDB 2484 and EMDB 5447) appear underlined.

It is clear that the pair of map (EMDB 2484) and particles from Bartesaghi provide the highest value of Q, but the difference with the maps EMDB 5018, EMDB 5019 and

EMDB 5462 is not large. Furthermore, when the Mao's particles are used maps EMDB 5018, EMDB 5019 and EMDB 5462 also provide high Q values. Our interpretation of this result comes from the high dependence of the particle alignment precision on the spatial map resolution. Here, it is important to make the distinction between FSC resolution, which is a measure of consistency between two maps at a given frequency, and the resolution understood in terms of the highest frequency (being signal or noise) that appear in a map. In Figure S.7 we show maps EMDB 5447, EMDB 5418 and EMDB 5019 low pass filtered to 20 Å resolution using in all cases the same low pass filter.

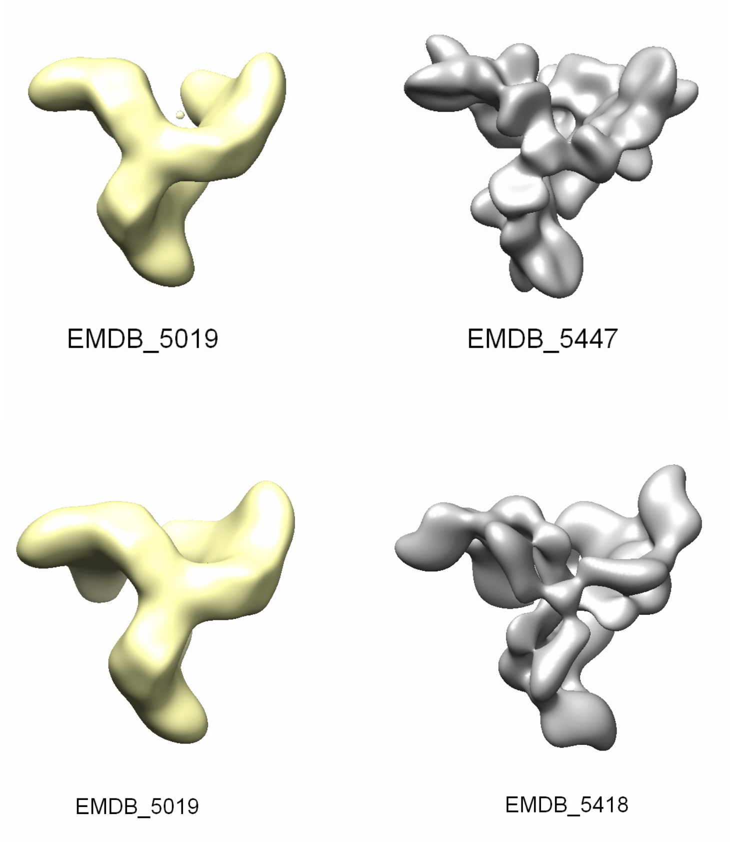

Figure S.7 Maps EMDB 5019, EMDB 5447 and EMDB 5418 filtered to 20 Å.

From the figures can be seen that the resolution of map EMDB 5019 is clearly larger than 20 Å as the resolution of maps EMDB 5418 and EMDB 5447 is of 20 Å. Similar

visual analysis can be done with maps EMDB 5018 and EMDB 5462 which also show larger resolutions than 20 Ǻ.
